# Biological Effect of Food for Special Medical Purposes (Nutramil^TM^ Complex) on Melanoma Cells in In Vitro Study

**DOI:** 10.3390/nu16244287

**Published:** 2024-12-12

**Authors:** Aneta Koronowicz, Katarzyna Krawczyk, Aleksandra Such, Ewelina Piasna-Słupecka, Mariola Drozdowska, Teresa Leszczyńska

**Affiliations:** Department of Human Nutrition and Dietetics, Faculty of Food Technology, University of Agriculture in Krakow, 30-149 Krakow, Poland; katarzyna.krawczyk@student.urk.edu.pl (K.K.); aleksandra.such@student.urk.edu.pl (A.S.); ewelina.piasna@urk.edu.pl (E.P.-S.); mariola.drozdowska@urk.edu.pl (M.D.); teresa.leszczyska@urk.edu.pl (T.L.)

**Keywords:** Nutramil^TM^ complex, FSMP, melanoma, apoptosis, cellular stress response

## Abstract

Background/Objectives: Melanoma malignum is considered the most dangerous form of skin cancer, characterized by the exceptional resistance to many conventional chemotherapies. The aim of this study was to evaluate the effect of Nutramil^TM^ Complex (NC)—Food for Special Medical Purpose (FSMP), on two types of melanoma cell lines, primary WM115 and malignant WM266-4. Methods: At 24 h after seeding, growth medium was replaced with a medium containing encoded treatments of NC or NC-CC (Nutramil^TM^ Complex without calcium caseinate) at various concentrations. Cells were treated for 24, 48, and 72 h. Results: Our results showed that Nutramil^TM^ Complex reduces proliferation of malignant melanoma WM266-4 cells but did not affect the proliferation of WM115 primary melanoma. This was followed by measured down-regulation of selected pro-survival proteins expression in WM266-4 cells, specifically ERK1/2, AKT-1, HSP27, Survivin, and TAK1. Interestingly, our results showed elevated levels of some pro-apoptotic proteins in both cell lines, including Bad, Smad2, p38MAPK, cleaved forms of Caspase-3/7, as well as cleaved PARP. Conclusions: Taken together, our results indicate that various melanoma cancer cell lines may respond in a different way to the same compound. They also suggest induction of apoptotic pathway by Nutramil^TM^ Complex as the most likely mechanism of its anticarcinogenic activity.

## 1. Introduction

Melanoma is one of the most malignant human cancers, with rising incidence rates worldwide, which accounted for 1.7% of all cancers in 2020 [1]. In the International Agency for Research on Cancer’s latest GLOBOCAN 2022 study, melanoma ranked 17th among the most common cancers worldwide and 22nd in terms of mortality [2]. Regarding the US, the American Cancer Society estimated 99.780 new cases and mortality at about 7.650 cancer deaths in the United States [3].

Melanoma cells are characterized by exceptional therapy resistance; thus, in addition to research on new pharmacological strategies, studies are carried out on cancer prevention, including the effect of diet. Results of those studies, mainly epidemiological, showed promising benefits of many dietary nutrients in melanoma chemoprevention [4].

In addition, malnutrition is often observed in patients with melanoma, which leads to a weakening of the body and a worsening prognosis for recovery. For this reason, the main task of special medical food, in this case Nutramil^TM^ Complex (NC), is to provide the patient with nutritional support, and the potential anti-cancer effect is an added value [5].

Nutramil™ Complex is a Food for Special Medical Purpose (FSMP) composed of all essential nutrients—proteins, carbohydrates, fats, vitamins, micro- and macroelements. It is designed to provide a nutritionally complete and balanced diet for people whose nutritional requirements cannot be met by normal foods. In Europe, the FSMP product composition and placing on the market is regulated by the REGULATION (EU) No. 609/2013, supplemented by the COMMISSION DELEGATED REGULATION (EU) 2016/128. Article 9 of the supplement makes each Member State solely responsible for the enforcement of the FSMP legislation. FSMP legislation itself does not impose a registration procedure on the food business operator, as required from medicinal products, but relies on the self-assessment that the product complies with regulations. Therefore, FSMP products are not subjected to preclinical and clinical studies.

Food products for Special Medical Purposes (FSMPs) are important in cancer therapy, especially in counteracting malnutrition and improving the quality of life of cancer patients. The use of FSMPs in enteral nutrition helps maintain body weight and improve caloric intake, which promotes therapy tolerance and quality of life, improves treatment outcomes, and reduces length of hospitalization [6]. In head and neck cancer patients undergoing radiation therapy, the use of FSMPs significantly improved energy intake and reduced weight loss compared to patients not following dietary recommendations [7]. A study in Romania indicated that cost and limited awareness of FSMP products are significant barriers for oncology patients. Better education and regulation of online sales could improve their availability and effective use [8].

As a result, available literature is very limited on FSMP. A rare example is our earlier work reporting the effect of Nutramil™ Complex (NC) on breast and prostate cancer cells; specifically, the induction of mitochondrial apoptotic pathway [9]. The aim of this study was to evaluate the effects of Nutramil^TM^ Complex (NC) and NC-CC (Nutramil^TM^ Complex without calcium caseinate) on two types of melanoma cell lines, primary WM115 and malignant WM266-4. Our objectives were to determine their time- and dose-dependent effect on cytotoxicity and cell proliferation. In addition, we determined the levels of expression of genes regulating the cell cycle and apoptosis, as well as the expression of selected pro-survival and pro-apoptotic proteins. Interestingly, our results showed two different responses of these cells to the applied product.

## 2. Materials and Methods

### 2.1. Testing Material

Nutramil^TM^ Complex (NC) was the testing material and its composition is described in our previous publication [9]. To determine the effect of complete compound on cancer cells, an incomplete Nutramil^TM^ Complex without calcium caseinate (NC-CC) was also investigated. We hypothesized that the complete formulation would have the greatest effect on cellular parameters. On the other hand, we would like to check if the protein exclusion would maintain the observed changes, which may be useful in a possible modification of the nutrient composition. All analyses were blanked. All samples were numerically coded and decoded at the end of this study.

### 2.2. Cell Cultures and Treatments

This research was conducted with use of human primary melanoma cell line WM115 (ATCC^®^ CRL-1675TM), human malignant melanoma cell line WM266-4 (ATCC^®^ CRL-1676TM), and human foreskin fibroblast cell line BJ (ATCC^®^ CRL-2522TM). Cells were cultured in appropriate medium with the addition of 10% FBS (Sigma-Aldrich, St. Louis, MO, USA) and under controlled conditions (temperature 37 °C and 5% CO_2_ atmosphere) according to the ATCC protocol.

Cancer cells were seeded in a density of 8 × 10^3^ cells per well in 96-well plates, 9 × 10^4^ cells per well in 12-well plates, and 2 × 10^5^ per well in 6-well plates. At 24 h after seeding, growth medium was replaced with a medium containing encoded treatments of NC or NC-CC at various concentrations. The final applied concentrations of NC and NC-CC were 1, 2, 3, 4, 5, and 10%. Then, the cells were incubated for 24, 48, and 72 h. Cells cultivated only in complete growth medium were used as a negative control. Staurosporine (Sigma-Aldrich, St. Louis, MO, USA) at final concentration at 1.5 μM was used as positive control for apoptosis assay. Staurosporine is characterized by strong and broad proapoptotic activity, so it is widely used in apoptosis research [10,11].

### 2.3. Cytotoxicity Assay

To evaluate cell cytotoxicity, the LDH Cytotoxicity Detection Kit (Roche, Basel, Switzerland) was used. This assay measures the activity of lactate dehydrogenase (LDH) released into the culture medium as a result of cell death. The procedure was carried out according to the manufacturer’s guidelines. Three independent experiments were conducted, each with 4–5 technical replicates.

### 2.4. Cell Proliferation Assay

Cell proliferation was assessed using the 5′-bromo-2′-deoxy-uridine (BrdU) Labeling and Detection Kit III (Roche, Basel, Switzerland) following the manufacturer’s instructions. All experiments were conducted in three independent runs, with measurements taken in triplicate. Results were normalized to the negative control (untreated cells), which was set as 100%.

### 2.5. RNA Isolation, RT Reaction and Real-Time PCR Analysis

Total RNA was isolated from cell lines using the Total RNA Mini Kit (A&A Biotechnology, Gdansk, Poland) following the protocol provided by the manufacturer. The concentration, purity, and quality of RNA were assessed using a µDrop Plate (Thermo Fisher Scientific, Waltham, MA, USA). Reverse transcription was carried out with the Maxima First Strand cDNA Synthesis Kit for RT-qPCR (Thermo Fisher Scientific, Waltham, MA, USA). Quantitative mRNA expression analysis was performed using TaqMan^®^ Array Human C-MYC and Apoptosis panels (Thermo Fisher Scientific, Waltham, MA, USA) in accordance with the manufacturer’s guidelines, utilizing the StepOnePlus™ System (Thermo Fisher Scientific, Waltham, MA, USA). Analysed genes included *AKT1*, *APAF1*, *BAD*, *BAX*, *BCL2*, *BID*, *CASP3*, *CASP8*, *CDKN2A*, *CYCS*, *FADD*, *FAS*, *FASLG*, *HRAS*, *IGF1*, *IGF1R*, *KRAS*, *MYC*, *NRAS*, *RRAS*, *TP53*, *YWHAB*, *YWHAE*, *YWHAG*, *YWHAH*, *YWHAQ*, *YWHAZ*. Results were normalized using at least two reference genes (*18S*, *GAPDH*, *HPRT1* or *GUSB*) and were calculated using the 2^−∆∆CT^ method [12].

### 2.6. Stress and Apoptosis Signaling Assay

Cell extracts were prepared and analyzed using the PathScan^®^ Stress and Apoptosis Signaling Antibody Array Kit (Chemiluminescent Readout #12856, Cell Signaling Technology, Danvers, MA, USA). Assay target proteins were P44/42 MAPK (ERK1/2) phosphorylation, Akt phosphorylation, Bad phosphorylation, HSP27 phosphorylation, Smad2 phosphorylation, p53 phosphorylation, p38 MAPK phosphorylation, SAPK/JNK phosphorylation, PARP cleavage, Caspase-3 cleavage, Caspase-7 cleavage, Ikβα total, Chk1 Ser345 phosphorylation, Chk2 phosphorylation, Ikβα phosphorylation, eIF2α phosphorylation, TAK1 phosphorylation, Survivin, and α-Tubulin as a reference protein. Images were acquired by briefly exposing the slide to standard chemiluminescent film. Densitometry analysis was performed using ImageJ software version 1.45s; NIH, Bethesda, MD, USA; http://imagej.nih.gov/ij. Results, expressed as mean ± SD, were normalized to the internal reference protein (α-Tubulin), with untreated negative control (UC) set at 100% expression.

### 2.7. Statistical Analysis

Statistical analysis of all experiments in melanoma and BJ cells was conducted using an independent samples *t*-test to compare unpaired means between two groups. *p* values less than 0.05 were considered as statistically significant.

Statistical analysis was conducted using Statistica version 12 (StatSoft, Tulsa, OK, USA). Each experiment was performed in three independent runs, with measurements taken in triplicate. The Shapiro–Wilk test was used to evaluate the normality of data distribution. Results are presented as mean ± standard deviation (SD). Statistical analysis of all experiments involving melanoma and BJ cells was performed using an independent samples *t*-test to compare the means of two unpaired groups. A *p*-value of less than 0.05 was considered statistically significant.

## 3. Results

### 3.1. Cytotoxicity

**Figure 1:**
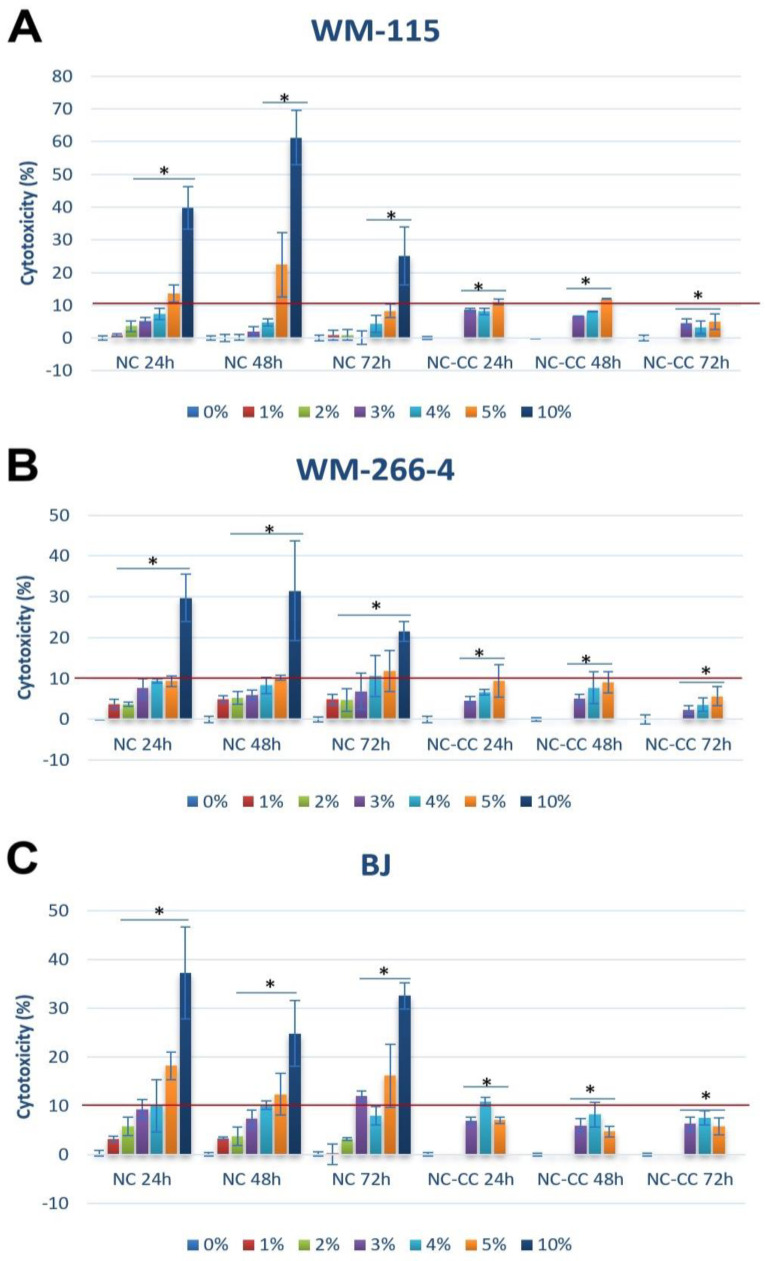
Cytotoxicity of NutramilTM Complex and NutramilTM Complex without calcium caseinate in human melanoma cell lines: (**A**) WM-115, (**B**) WM-266-4, and (**C**) BJ normal fibroblast cell line. Cells were exposed to 1–10% concentrations of Nutramil^TM^ Complex (NC) or Nutramil^TM^ Complex without calcium caseinate (NC-CC) for 24, 48, and 72 h. Data are presented as mean ± SD for n = 15. Statistical significance was determined by a *t*-test; * denotes *p* < 0.05 compared to the untreated control (UC).

Nutramil™ Complex showed strong cytotoxic effects on both examined melanoma cell lines (WM266-4 and WM115), as well as non-neoplastic BJ cells (Figure 1).

Cytotoxicity was dose- and time-dependent and showed a similar trend for both NC-CC (NutramilTM Complex without calcium caseinate) and NC treatment. Application of Cytotoxicity Detection Kit LDH (Roche, Warszawa, Poland) showed that 4% treatment did not initiate necrosis (Figure 1A–C). Based on the results, 4% concentration was chosen for further studies as the inhibitory concentration for 10% cytotoxicity (EC10).

### 3.2. Cell Proliferation

**Figure 2:**
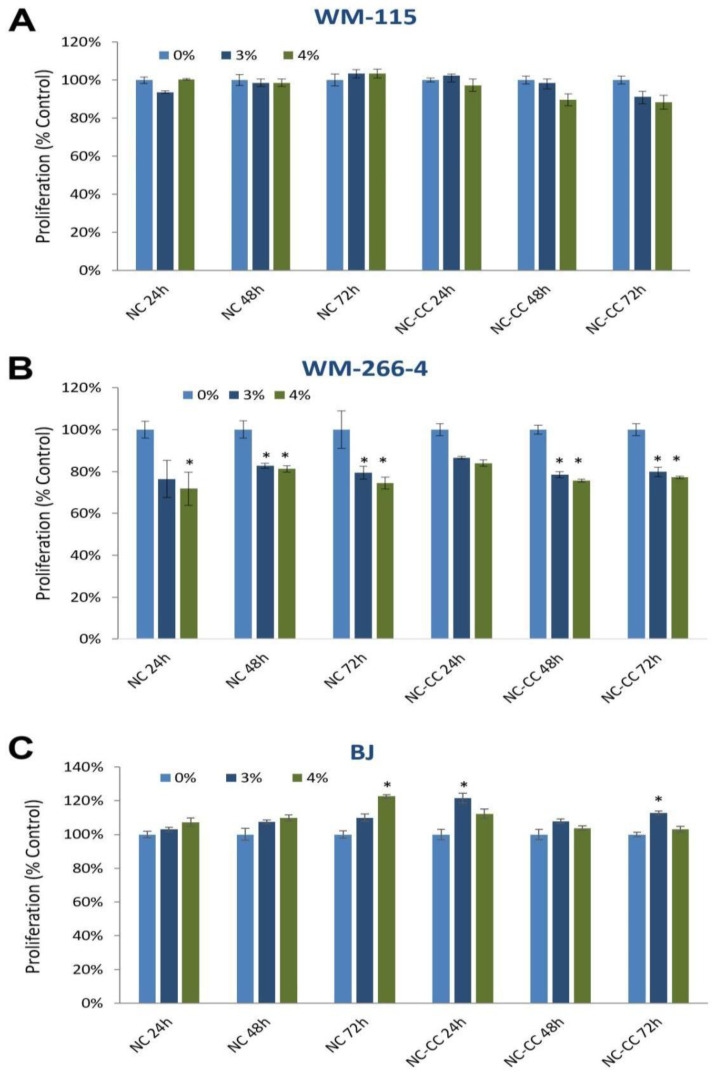
The effect of Nutramil^TM^ Complex and Nutramil^TM^ Complex without calcium caseinate on proliferation in human melanoma cell lines (WM-115, WM-266-4) and normal fibroblast cell line (BJ). Cells WM-115 (**A**), WM266-4 (**B**), and BJ (**C**) were treated with Nutramil^TM^ Complex (NC) or Nutramil^TM^ Complex without calcium caseinate (NC-CC) at concentration 0, 3, 4% for 24, 48, and 72 h. Data are presented as mean ± SD for n = 12, normalized to the untreated control (UC) set as 100%. Statistical significance was determined by a *t*-test; * denotes *p* < 0.05 compared to UC.

The BrdU test demonstrated that Nutramil™ Complex reduced the proliferation of WM266-4 cell line to approximately 75–80% of the negative control (Figure 2B) but did not affect WM115 melanoma cells (Figure 2A). In contrast, NC stimulated proliferation of normal BJ cell line (Figure 2C). Treatment with NC-CC cells showed similar effects as NC on studied cell lines (Figure 2A–C).

### 3.3. mRNA Expression of Genes Associated with Cell Cycle and Apoptosis

The expression of genes regulating cell cycle and apoptosis was investigated with TaqMan^®^ Array C-MYC and Apoptosis Kit. Results are presented in Table 1.

For WM-115 primary melanoma cells, results showed up-regulation of many pro-apoptotic genes, including *APAF1*, *BAD*, *BAX*, *BID*, *CASP3*, *CASP8*, *CASP9*, *CYCS*, *FADD*, and down-regulation of pro-survival genes *BCL-2*, *HRAS*, *IGF1*, *KRAS*, *MYC*, and *YWHA* family.

For WM266-4 melanoma cells, results showed up-regulation of pro-apoptotic *BAD*, *BAX*, *BID*, *CASP3*, *CASP9*, *FADD*, *FAS*, and *TP53*, and down-regulation of *APAF1* and *CYCS.* Pro-survival genes, including *AKT1*, *BCL-2*, *HRAS*, *IGF1*, *IGF1R*, *KRAS*, *NRAS*, *MYC*, and genes from the *YWHA* family, showed a reduction in their mRNA levels after NC treatment.

Results for treatment with NC-CC showed similar trends (Table 1).

### 3.4. Expression of Proteins Involved in Cellular Stress and Apoptosis Signaling

**Figure 3:**
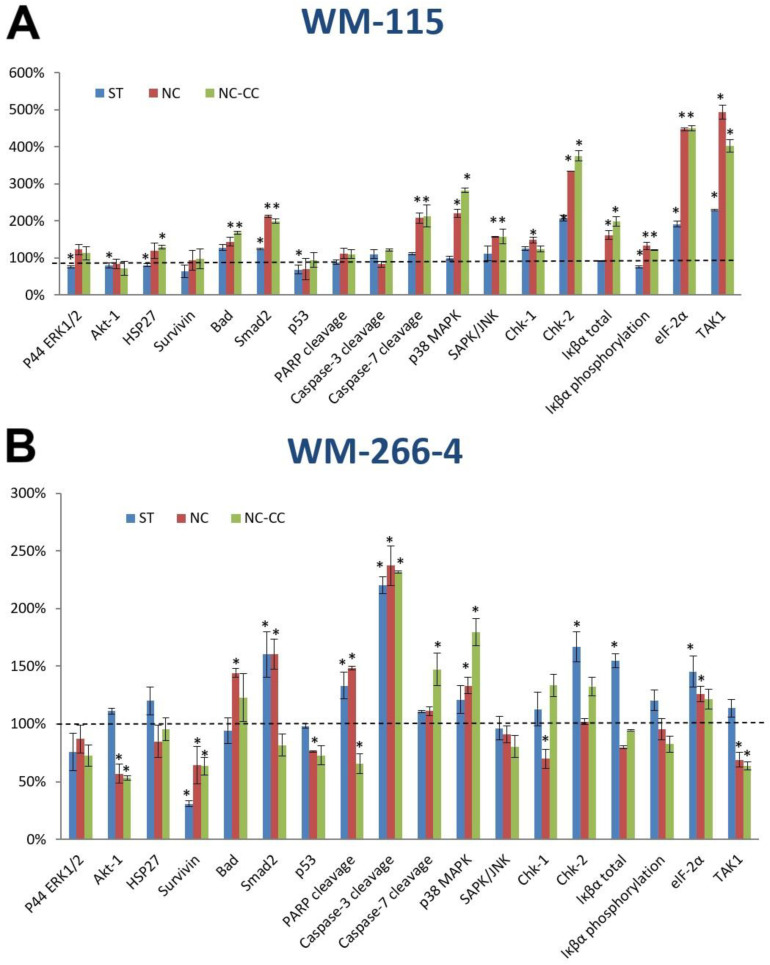
The effect of Nutramil^TM^ Complex and Nutramil^TM^ Complex without calcium caseinate on expression of stress and apoptosis proteins in melanoma cells (WM-115, WM-266-4). Cells WM-115 (**A**) and WM266-4 (**B**) were treated for 48 h with 4% of Nutramil ^TM^ Complex (NC) or Nutramil ^TM^ Complex without calcium caseinate (NC-CC). Staurosporine (ST; 1.5 μM concentration) was used as positive control. The results are presented as mean ± SD, normalized to the internal reference protein (α-Tubulin), with the untreated control (UC) set as 100% expression. Statistical significance was determined using a *t*-test; * indicates *p* < 0.05 compared to UC. Gene symbols and names: P44/42 MAPK (ERK1/2) phosphorylation (Thr202/Tyr204), Akt-1 phosphorylation (Ser473), Bad phosphorylation (Ser136), HSP27 phosphorylation (Ser82), Smad2 phosphorylation (Ser465/467), p53 phosphorylation (Ser15), p38 MAPK phosphorylation (Thr180/Tyr182), SAPK/JNK phosphorylation (Thr183/Tyr185), PARP cleavage (Asp214), Caspase-3 cleavage (Asp175), Caspase-7 cleavage (Asp198), IkB total, Chk-1 phosphorylation (Ser345), Chk-2 phosphorylation (Thr68), IkBα phosphorylation (Ser32/36), eIF2a phosphorylation (Ser51), TAK1 phosphorylation (Ser412), Survivin total.

Results of the PathScan^®^ Stress and Apoptosis Signaling Antibody Array analysis for WM115 and WM266-4 are presented in Figure 3A,B, respectively.

Treatment of WM115 with NC did not significantly reduce levels of pro-survival proteins, including ERK1/2, AKT-1, HSP27, and Survivin; however, it significantly increased levels of phospho-BAD (128% of UC) as well as the cleaved form of Caspase -7 (200% of UC), Smad2 tumor suppressor (211% of UC), P38 MAPK (220% of UC), and SAPK/JNK (157% of UC). The levels of p53, Caspase-3, and PARP cleavage were not significantly affected. Expression of Chk-1 and Chk-2, total and phosphorylated Iκβα, eIF-2α, as well as TAK1 were significantly increased to the following (148%, 330%, 161%, 132%, 447%, 493% of UC, respectively) (Figure 3A).

Treatment of WM266-4 with NC significantly affected pro-survival proteins, including AKT-1 (57% of UC), Survivin (64% of UC), and TAK1 (69% of UC). Levels of ERK1/2 and HSP27 showed an insignificant reduction. Among the pro-apoptotic proteins, results showed an increase in levels of Bad, Smad2, p38 MAPK, cleaved forms of Caspase-3 and -7, as well as cleaved PARP (144%, 160%, 134%, 237%, 111%, 148% of UC, respectively). In addition, Chk-1 levels were significantly reduced to 70% of UC, while Chk-2 remained unchanged. Expression of the p53 suppressor was reduced (76% of UC). The SAPK/JNK, Κβα-phospho and total Iκβα showed a decrease while the eIF-2α protein level was elevated to 126% of UC (Figure 3B).

For NC-CC, results for the majority of tested proteins were consistent with the results observed for the NC treatment (Figure 3A,B).

## 4. Discussion

Available epidemiological research indicates the importance of diet in reducing the risk and progression of melanoma. Anti-cancer properties are primarily expected from products with a strong antioxidant and anti-inflammatory potential, associated with the ability to reverse the oxidative damage caused by UV light [13]. On the other hand, main therapeutic strategies are focused on induction of apoptosis and suppression of survival pathways [14].

It should be noted that the main purpose of Food for Special Medical Purposes in cancer is primarily nutritional support for the patient. Potential anti-cancer activity should be considered as an added value in this case. We assume that NC and NC-CC ingredients (Table S1) that can inhibit malignant melanoma proliferation include medium-chain fatty acids which have been shown to enhance immune responses and induce apoptosis in cancer cells through activation of the EGFR/ERK/AP1 signaling pathway [15]. In addition, there are other bioactive components (vitamins and minerals) in the formulation that also contribute to suppressing cell proliferation by modulating oxidative stress and affecting cancer-related gene expression [16].

Our data showed that NC reduced proliferation of malignant melanoma WM266-4 cells but did not affect the proliferation of WM115 primary melanoma (Figure 2). Initially, both melanoma lines showed a measurable down-regulation of most of the analyzed pro-survival genes expression at the mRNA level (Table 1). However, these results were not confirmed for WM115 cells at the protein expression level (Figure 3A), which is consistent with the observed lack of changes in WM115 cell proliferation (Figure 2A). It should be emphasized that changes at the transcription level are not always reflected in protein expression [17]. Differences in response to NC treatment between primary (WM115) and metastatic (WM266-4) melanoma cells may indeed be due to their different stages. Metastatic WM266-4 cells have higher activity of enzymes related to cysteine metabolism and antioxidant defense mechanisms, such as 3-mercaptopyrate sulfurtransferase (MPST), which affects their sensitivity to oxidative stress and bioactive components [18]. In addition, the altered apoptotic response in WM266-4, incorporating TRAIL-DR5 mechanisms and specific programs of activated autophagy, may also explain the differences in response to therapy [19].

Measured protein expression in WM266-4 cells confirmed down-regulation of pro-survival ERK1/2, AKT-1, HSP27, Survivin, and TAK1 (Figure 3) and up-regulation of pro-apoptotic Bad, Smad2, p38 MAPK, cleaved forms of Caspase-3 and -7, as well as cleaved PARP (Figure 3B), resulting in decreased proliferation and induction of apoptosis (Figure 2A). Interestingly, similarly to WM266-4 cells, WM-115 cells showed a significant increase in levels of some pro-apoptotic proteins, including phospho-BAD, cleaved form of Caspase -7, P38 MAPK, and SAPK/JNK as well as Smad2 tumor suppressor (Figure 3A). The sensitivity of various melanoma cancer cell lines to the same compound may differ, depending on the distinctive mechanism of formation and progression. The speed of cell division and their translational activity are mentioned as the primary determinants [20]. The resistance of the tumor may also result from the decreased ability to undergo spontaneous apoptosis. Available research indicates that phenotype changes occurring during cancer development may play a crucial role in developing resistance to applied pro-apoptotic factors [21]. Our proliferation data would confirm that hypothesis as both NC-treated melanoma cell lines showed similar trends in pro-apoptotic protein expression, but they manifested a different response. In addition, our results also highlight the complexity of mechanisms and interactions that must be triggered to regulate growth and death of cancer cells.

Literature suggests that in human melanomas, two major signaling pathways RAS/RAF/MEK/ERK and the PI3K/Akt are constitutively activated through genetic mutations. It also suggests the influence of regulatory proteins of the Bcl-2 family [22,23].

The RAS/RAF/MEK/ERK signaling pathway is an important regulator of cell growth and survival and it has been reported to be activated in about 90% of human melanomas [24]. Of all RAS isoforms (HRAS, KRAS, and NRAS in humans), the most common mutation in melanomas occurs in the NRAS gene, while in other types of human cancers, mutations are more frequent in the KRAS gene [25]. Some melanomas exhibit an excessive activation of heat shock proteins, such as HSP27. HSP27 increases the resistance of melanoma cancer cells to apoptosis by binding cytochrome c and preventing the activation of caspase-9 and caspase-3 [26]. HSP27 also accelerates the proteolysis of p27 (CDK inhibitor), which prevents cell cycle arrest in the G1 phase [27]. HSP27 is also associated with the promotion of the proteasomal degradation of Iκβα—the inhibitor of the NF-κβ transcription factor. On the other hand, the presence of the phosphorylated form of Iκβα indicates degradation of the NF-κβ/Iκβ complex and subsequent translocation of NF-κβ to the nucleus, where it can activate many anti-apoptotic genes [9]. Treatment of WM-266-4 cells with the Nutramil^TM^ Complex had no significant effect on the level of the phosphorylated form of Iκβα. It also did not increase the level of the non-phosphorylated Iκβα, capable of binding to NF-κβ (Figure 3B). However, our results showed a reduced level of TAK1 protein, which may stimulate the phosphorylation of the Iκβα protein [28].

PI3K/Akt signaling was found dysregulated in over 50% of melanomas [29]. The PI3K/Akt signaling cascade is activated via the IGF growth factors’ paracrine/autocrine signal. Active RAS induces membrane translocation and activation of PI3K leading to PIP2 phosphorylation to PIP3 and activation of Akt protein. Akt effectors promote cell survival, proliferation, and invasion [30], including activation of NF-κB transcription factor [25], promoting expression of pro-survival proteins from the Bcl-2 family and inhibiting expression of pro-apoptotic Bad [31,32]. Nutramil^TM^ Complex had a down-regulating effect on the expression of *AKT1* gene and its protein in WM266-4 cells, while it had no significant effect in WM-115 cells (Table 1; Figure 3). However, down-regulation of *BCL-2* gene expression and increased expression of pro-apoptotic genes, including *BAD*, *BID*, and *BAX* were observed in both cell lines (Table 1). In addition, an increased level of Bad protein was also measured in both cell lines (Figure 3). Our results also showed an elevated level of phosphorylated kinases MAP p38 MAPK and SAPK/JNK MAP in both melanoma cell lines after NC treatment (Figure 3). The literature indicates that activation of p38 MAPK and SAPK/JNK MAP kinases occurs via a dual phosphorylation mechanism in response to cellular stressors and leads to the cell-cycle arrest and apoptosis induction. In addition, it is suggested that SAPK/JNK may contribute to the activation of both apoptotic pathways, the extrinsic and the mitochondrial-dependent pathway. In order to induce cell death, these mechanisms may interact or act independently [9], which finds confirmation in measured up-regulated expression of *FADD*, *FAS*, and *CASP8* genes (Table 1).

In contrast to many other cancers, the p53 suppressor gene is rarely mutated in melanoma [29] but its functional attenuation is needed for tumor development [33]. Our results showed an increased level of *TP53* gene mRNA in both NC-treated melanoma cell lines (Table 1); however, WM266-4 also showed a reduced level of p53 protein (Figure 3). This indicates potentially p53-independent induction of apoptosis in this cell line. These results are consistent with other studies on human melanoma cells that showed potential degradation of p53 protein rather than its accumulation [18]. In addition, recently, the essential p53 inhibitor Mdmx was determined to be frequently over-expressed in melanoma. Mdmx displays both p53-dependent and p53-independent oncogenic effects needed for melanoma growth [33].

*APAF-1* gene dysregulation is often indicated (42%) as one of the factors inhibiting the apoptotic process in melanoma cells; namely, by directly preventing caspase-9 activation and initiation of the protease cascade [34,35]. Our results showed that treatment of both melanoma lines with Nutramil^TM^ Complex inreased the expression of *APAF-1* as well as *CASP 9* and *CASP 3* (Table 1). However, simultaneous increase in the protein levels of apoptotic markers, including the cleaved form of caspase-3 and its substrate PARP, was observed only in WM266-4 cells (Figure 3). Similarly, only the WM266-4 cell line showed a reduction in the level of Survivin (Figure 3), a protein highly expressed in most cancer cells. Survivin inhibits the caspase activation in tumor cells, thereby suppressing their apoptosis and promoting cell proliferation [36,37,38]. It has been shown that by inhibiting Survivin expression via various mechanisms, including blocking its transcription, it increased the sensitivity of various cancer cells, including melanoma, to some chemotherapeutic agents [39,40]. For this reason, Survivin is considered an independent marker associated with poor prognosis [21,41]. Taken together, measured gene and protein expression supports presented proliferation results in the examined cell lines (Figure 2). In the WM115 cells, despite the observed activation of some pro-apoptotic genes, NC did not cause the death of these cells. On the other hand, the reduction in WM266-4 cell line proliferation was associated both with the down-regulation of pro-survival genes and the induction of apoptosis. Available literature indicates that dysregulation of apoptotic processes in melanoma cells are primarily associated with an impaired mitochondrial-dependent pathway. Although our results do not include the full set of proteins involved in this process, measured changes in expression of *BCL-2* genes, up-regulation of *APAF-1*, and reduced Survivin expression (Table 1, Figure 3) support this hypothesis. In addition, our previous study showed a similar effect of this FSMP on the induction of the intrinsic pathway of apoptosis in breast and prostate carcinoma cells [9]. Taken together, Nutramil^TM^ Complex, as a Food for Special Medical Purposes, may support the nutritional treatment of oncological patients. In addition, in the current and previous study, we presented its cytotoxic effect against cancer cells. This effect on reducing the growth of cancer cells, described in detail in our earlier publication, may be associated with the product composition, including the contribution and chemical form of the particular macro- and microelements [9].

Differences between mRNA and protein levels result from multiple overlapping processes, including regulation at the level of transcription, mRNA stability, translation, and protein degradation. These mechanisms allow cells to precisely control gene expression in response to changing needs and environmental conditions. In WM115, the expression of most pro-apoptotic genes (e.g., *APAF1*, *BAD*, *BAX*, *BID*, *CASPs*) increased at the mRNA level, but these changes were not fully reflected in the levels of the tested proteins both pro-survival (Akt-1, ERK1/2, Survivin) and pro-apoptotic (e.g., Parp, Caspase 3). This may explain the lack of effect on cell proliferation in this cell line. In WM266-4, a decrease in the expression of pro-survival genes was confirmed at both the mRNA and protein levels, resulting in decreased proliferation. Translational and post-translational mechanisms influence the differences between mRNA and protein levels. For example, proteins such as HSP27 can alter resistance to apoptosis, and their regulation does not necessarily correlate with mRNA levels. Differences in the activation of MAPK kinases, such as p38 MAPK, and the presence of cleaved forms of Caspases (e.g., Caspase-3 and Caspase-7) also influence the final biological effect. Variable levels of mRNA and protein expression emphasize that differences in the response of different cell lines to the same agents are due to their specific translational activity and ability to induce apoptotic mechanisms.

The results obtained from in vitro studies provide valuable information on the potential mechanisms of action and therapeutic effects of substances, but it has its limitations. First of all, in vitro studies do not completely reflect the complexity of interactions between tumor cells and the surrounding microenvironment. In addition, the results obtained in cell cultures may not take into account the effects on other tissues, immune systems, or metabolism, which are important for anti-cancer therapies [42,43]. In vivo validation, including animal model studies, is essential to confirm the efficacy and therapeutic safety of test substances in living organisms, allowing assessment of their pharmacokinetics, potential side effects, and interactions with biological systems [44]. Further studies in in vivo models may also help to understand mechanisms that may differ from those observed in cell cultures.

## 5. Conclusions

In conclusion, it should be emphasized that the results presented in the current paper are one of the very few investigating the effect of the selected FSMP product, Nutramil^TM^ Complex, on the proliferation of melanoma cells. Results obtained from the WM266-4 line cells are consistent with our previous findings in breast and prostate cancer lines, which confirms the beneficial effect of the product. On the other hand, the observed differences in response to the NC treatment between two studied melanoma line cells may result from their different sensitivity. Although our experiments are limited to the in vitro model and require verification in vivo, we find those tests necessary according to the 3R principle (Replacement, Reduction, and Refinement). Together, they serve as a valuable and multifaceted source of information and provide justification for further research.

## Figures and Tables

**Table 1:** The effect of Nutramil^TM^ Complex and Nutramil^TM^ Complex without calcium caseinate on mRNA expression of genes in human melanoma WM-115 and WM-266-4 cells lines.

Gene Symbol	WM-115				WM266-4			
	NC vs. UC		NC-CC vs. UC		NC vs. UC		NC-CC vs. UC	
	FC Value	Adjusted *p*-Values	FC Value	Adjusted *p*-Values	FC Value	Adjusted *p*-Values	FC Value	Adjusted *p*-Values
Pro-apoptotic genes								
*APAF1*	↑4.52 *	1.1 × 10^−7^	↑4.73 *	0.00003	↓−4.56 *	0.00001	↓−2.69 *	0.00010
*BAD*	↑1.59 *	0.01212	↑1.25 *	0.00007	↑6.20 *	0.01623	↑3.62 *	0.00128
*BAX*	↑1.37 *	0.00064	1.44	0.07656	↑2.12 *	0.00005	1.29	0.05086
*BID*	↑1.78 *	0.02290	↑1.25 *	0.00552	↑2.58 *	0.00013	1.74	0.12706
*CASP3*	↑2.42 *	0.00017	↑2.79 *	5.0 × 10^−7^	↑4.00 *	0.00017	↑3.27 *	0.00005
*CASP8*	↑4.01 *	0.00015	↑5.53 *	0.00007	↓−1.55 *	0.00001	↓−2.28 *	0.00013
*CASP9*	↑2.91 *	0.00148	↑1.40 *	0.00752	↑4.80 *	0.00148	↑2.93 *	0.00007
*CYCS*	↑1.73 *	0.03390	↑1.48 *	0.00641	↓−2.06 *	0.00295	↓−2.76 *	0.00006
*FADD*	1.20	0.08572	↑1.47 *	0.01262	↑3.16 *	0.00008	1.87	0.163556
*FAS*	1.01	0.3740	↑1.12 *	2.80 × 10^−5^	1.12	0.37390	↑1.34 *	0.00006
*TP53*	1.02	0.09595	↑1.49 *	0.02467	↑1.72 *	0.00001	1.28	0.06596
Pro-survival genes								
*AKT1*	1.05	0.28798	1.18	0.12187	↓−1.97 *	0.00014	↓−1.42 *	0.02336
*BCL2*	−1.36	0.19346	↓−1.37 *	0.00859	↓−1.57 *	0.00022	↓−2.53 *	0.00023
*HRAS*	↓−1.82 *	0.02360	−1.69	0.51894	↓−2.38 *	0.00033	↓−1.64 *	0.00358
*IGF1*	↓−3.04 *	0.04336	1.01	0.11020	↓−7.70 *	0.00003	−2.16	0.08021
*IGF1R*	↑1.56 *	0.00003	↑1.27 *	0.00040	−2.01	0.43357	↓−1.43 *	0.00005
*KRAS*	↓−2.28 *	0.00004	↓−1.18 *	0.00008	↓−2.67 *	0.00004	↓−3.29 *	0.00009
*MYC*	↓−2.55 *	0.00366	↑1.12 *	0.00006	↓−1.10 *	0.00006	↓−1.23 *	0.03746
*NRAS*	↑1.16 *	0.00015	↑1.18 *	0.00018	−3.43 *	0.00001	↓−3.70 *	0.00001
*RRAS*	↑1.34 *	0.01637	−1.60	0.60253	↑1.17 *	0.00007	−1.15	0.05788
*YWHA* family genes								
*YWHAB*	↓−2.27 *	0.00027	↑1.04 *	0.00038	↓−2.46 *	0.00001	↓−1.27 *	0.00014
*YWHAE*	↓−1.57 *	0.00001	↓−1.29 *	0.00003	↓−2.92 *	0.00001	↓−3.86 *	0.00001
*YWHAG*	↓−1.85 *	0.01506	↓−1.29 *	0.00280	↓−1.08 *	0.00008	↓−1.56 *	0.00496
*YWHAH*	↓−1.73 *	0.00376	↓−1.38 *	0.00007	↓−1.88 *	0.00009	↓−1.55 *	0.00754
*YWHAQ*	1.01	0.10424	↓−1.10 *	0.00007	↓−2.35 *	0.00001	↓−4.10 *	0.00001
*YWHAZ*	↓−1.10 *	0.00029	↑1.10 *	0.00081	↓−2.38 *	0.00002	↓−4.10 *	0.00082

Cells were treated for 48h with 4% of Nutramil^TM^ Complex (NC) or Nutramil^TM^ Complex without calcium caseinate (NC-CC). Statistical significance of treatment: * vs. untreated control (UC) when *p* < 0.05. Ns: no signal. ↑: up-regulation. ↓: down-regulation. Gene symbols and names: *AKT1*, Serine/Threonine Protein Kinase 1; *APAF1*, Apoptotic Peptidase Activating Factor 1; *BAD*, Bcl2-Associated Death Promoter; *BAX*, BCL2 Associated X Protein, Apoptosis Regulator; *BID*, BH3 Interacting Domain Death Agonist; *BCL2*, Protein, Apoptosis Regulator; *CASP3*, Caspase 3; *CASP8*, Caspase 8; *CASP9*, Caspase 9; CYCS, Cytochrome C; *FADD*, Fas-Associated Death Domain; *FAS*, Fas Cell Surface Death Receptor; *HRAS*, HRas Proto-Oncogene, GTPase; *IGF1*, Insulin Like Growth Factor 1; *IGF1R*, Insulin Like Growth Factor 1 Receptor; *KRAS*, KRAS Proto-Oncogene, GTPase; *MYC*, MYC Proto-Oncogene, BHLH Transcription Factor; *NRAS*, NRAS Proto-Oncogene, GTPase; *RRAS*, Related RAS Viral (R-Ras) Oncogene Homolog; *TP53*, Tumor Protein P53; *YWHAB*, Tyrosine 3-Monooxygenase/Tryptophan 5-Monooxygenase Activation Protein Beta; *YWHAE*, Tyrosine 3 Monooxygenase/Tryptophan 5-Monooxygenase Activation Protein Epsilon; *YWHAG*, Tyrosine 3 Monooxygenase/Tryptophan 5-Monooxygenase Activation Protein Gamma; *YWHAH*, Tyrosine 3 Monooxygenase/Tryptophan 5-Monooxygenase Activation Protein Eta; *YWHAQ*, Tyrosine 3 Monooxygenase/Tryptophan 5-Monooxygenase Activation Theta; *YWHAZ*, Tyrosine 3 Monooxygenase/Tryptophan 5-Monooxygenase Activation Zeta.

## Data Availability

The original contributions presented in this study are included in the article/Supplementary Materials. Further inquiries can be directed to the corresponding author.

## Supplementary Materials

The following supporting information can be downloaded at: https://www.mdpi.com/article/10.3390/nu16244287/s1, Tabel S1. Composition of NutramilTM Complex as Food for Special Medical Purpose [9].

## References

[1] Sung H., Ferlay J., Siegel R.L., Laversanne M., Soerjomataram I., Jemal A., Bray F. (2021). Global Cancer Statistics 2020: GLOBOCAN Estimates of Incidence and Mortality Worldwide for 36 Cancers in 185 Countries. CA Cancer J. Clin..

[2] Globocan (2022). Global Cancer Observatory. https://gco.iarc.who.int/media/globocan/factsheets/cancers/16-melanoma-of-skin-fact-sheet.pdf.

[3] Siegel R.L., Miller K.D., Fuchs H.E., Jemal A. (2022). Cancer statistics. CA Cancer J. Clin..

[4] Tong L.X., Young L.C. (2014). Nutrition: The future of melanoma prevention?. J. Am. Acad. Dermatol..

[5] Arends J. (2023). Malnutrition in cancer patients: Causes, consequences and treatment options. Eur. J. Surg. Oncol. (EJSO).

[6] Frydrych A., Krośniak M., Jurowski K. (2023). The Role of Chosen Essential Elements (Zn, Cu, Se, Fe, Mn) in Food for Special Medical Purposes (FSMPs) Dedicated to Oncology Patients—Critical Review: State-of-the-Art. Nutrients.

[7] Surwiłło-Snarska A., Kapała A., Szostak-Węgierek D. (2024). Assessment of the Dietary Intake Changes in Patients with Head and Neck Cancer Treated with Radical Radiotherapy. Nutrients.

[8] Chereches M.C., Popa C.O., Finta H. (2024). The dynamics of food for special medical purposes (FSMPs) utilization in cancer care: From doctor recommendations to online pharmacy procurement. Front. Pharmacol..

[9] Koronowicz A.A., Drozdowska M., Wielgos B., Piasna-Słupecka E., Domagała D., Dulińska-Litewka J., Leszczyńska T. (2018). The effect of “NutramilTM Complex”, food for special medical purpose, on breast and prostate carcinoma cells. PLoS ONE.

[10] Belmokhtar C.A., Hillion J., Ségal-Bendirdjian E. (2001). Staurosporine induces apoptosis through both caspase-dependent and caspase-independent mechanisms. Oncogene.

[11] Antonsson A., Persson J.L. (2009). Induction of Apoptosis by Staurosporine Involves the Inhibition of Expression of the Major Cell Cycle Proteins at the G2/M Checkpoint Accompanied by Alterations in Erk and Akt Kinase Activities. Anticances Res..

[12] Livak K.J., Schmittgen T.D. (2001). Analysis of relative gene expression data using real-time quantitative PCR and the 2^−ΔΔCT^ Method. Methods.

[13] Yang K., Fung T.T., Nan H. (2018). An Epidemiological Review of Diet and Cutaneous Malignant Melanoma. Cancer Epidemiol. Biomark. Prev..

[14] Eberle J., Kurbanov B.M., Hossini A.M., Trefzer U., Fecker L.F. (2007). Overcoming Apoptosis Deficiency of melanoma—Hope for New Therapeutic Approaches. Drug Resist. Updat..

[15] Roopashree P., Shetty S.S., Kumari N.S. (2021). Effect of medium chain fatty acid in human health and disease. J. Funct. Food.

[16] Fagbohun O.F., Gillies C.R., Murphy K.P.J., Rupasinghe H.P.V. (2023). Role of Antioxidant Vitamins and Other Micronutrients on Regulations of Specific Genes and Signaling Pathways in the Prevention and Treatment of Cancer. Int. J. Mol. Sci..

[17] Master A., Nauman A. (2014). Molecular mechanisms of protein biosynthesis initiation--biochemical and biomedical implications of a new model of translation enhanced by the RNA hypoxia response element (rHRE). Postep. Biochem..

[18] Rydz L., Wróbel M., Janik K., Jurkowska H. (2023). Hypoxia-Induced Changes in L-Cysteine Metabolism and Antioxidative Processes in Melanoma Cells. Biomolecules.

[19] Giannopoulou A.F., Velentzas A.D., Anagnostopoulos A.K., Agalou A., Papandreou N.C., Katarachia S.A., Koumoundourou D.G., Konstantakou E.G., Pantazopoulou V.I., Delis A. (2021). From Proteomic Mapping to Invasion-Metastasis-Cascade Systemic Biomarkering and Targeted Drugging of Mutant BRAF-Dependent Human Cutaneous Melanomagenesis. Cancers.

[20] McConkey D.J., Zhu K. (2008). Mechanisms of proteasome inhibitor action and resistance in cancer. Drug Resist. Updat..

[21] Cichorek M., Kozłowska K., Wachulska M., Zielińska K. (2006). Spontaneous apoptosis of melanotic and amelanotic melanoma cells in different phases of cell cycle: Relation to tumor growth. Folia Histochem. Cytobiol..

[22] Pokrywka M., Litynska A. (2012). Targeting the melanoma. Postepy Biol. Komorki.

[23] Blokx W.A.M., Van Dijk M.C.R.F., Ruiter D.J. (2009). Molecular cytogenetics of cutaneous melanocytic lesions—Diagnostic, prognostic and therapeutic aspects. Histopathology.

[24] Gray-Schopfer V., Wellbrock C., Marais R. (2007). Melanoma biology and new targeted therapy. Nature.

[25] Sullivan R.J., Atkins M.B. (2009). Molecular-targeted therapy in malignant melanoma. Expert Rev. Anticancer. Ther..

[26] Sidor-Kaczmarek J., Cichorek M., Spodnik J.H., Wójcik S., Moryś J. (2017). Proteasome inhibitors against amelanotic melanoma. Cell Biol. Toxicol..

[27] Parcellier A., Brunet M., Schmitt E., Col E., Didelot C., Hammann A., Nakayama K.I., Khochbin S., Solary E., Garrido C. (2006). HSP27 favors ubiquitination and proteasomal degradation of p27 ^Kip1^ and helps S-phase re-entry in stressed cells. FASEB J..

[28] Okada M., Matsuzawa A., Yoshimura A., Ichijo H. (2014). The Lysosome Rupture-activated TAK1-JNK Pathway Regulates NLRP3 Inflammasome Activation. J. Biol. Chem..

[29] Dahl C., Guldberg P. (2007). The genome and epigenome of malignant melanoma. Apmis.

[30] Yajima I., Kumasaka M.Y., Thang N.D., Goto Y., Takeda K., Yamanoshita O., Iida M., Ohgami N., Tamura H., Kawamoto Y. (2011). RAS/RAF/MEK/ERK and PI3K/PTEN/AKT Signaling in Malignant Melanoma Progression and Therapy. Dermatol. Res. Pract..

[31] Krześlak A. (2010). Akt kinase: A key regulator of metabolism and progression of tumors. Adv. Hyg. Exp. Med..

[32] Koronowicz A.A., Banks P., Domagała D., Master A., Leszczyńska T., Piasna E., Marynowska M., Laidler P. (2016). Fatty acid extract from CLA-enriched egg yolks can mediate transcriptome reprogramming of MCF-7 cancer cells to prevent their growth and proliferation. Genes Nutr..

[33] Jochemsen A.G. (2014). Reactivation of p53 as therapeutic intervention for malignant melanoma. Curr. Opin. Oncol..

[34] Kyrgidis A., Tzellos T.-G., Triaridis S. (2010). Melanoma: Stem cells, sun exposure and hallmarks for carcinogenesis, molecular concepts and future clinical implications. J. Carcinog..

[35] Ko J.M., Velez N.F., Tsao H. (2010). Pathways to Melanoma. Semin. Cutan. Med. Surg..

[36] Wheatley S.P., McNeish I.A. (2005). Survivin: A Protein with Dual Roles in Mitosis and Apoptosis. Int. Rev. Cytol..

[37] O’Driscoll L., Linehan R., Clynes M. (2003). Survivin: Role in Normal Cells and in Pathological Conditions. Curr. Cancer Drug Targets.

[38] Tamm I., Wang Y., Sausville E., A Scudiero D., Vigna N., Oltersdorf T., Reed J.C. (1998). IAP-family protein survivin inhibits caspase activity and apoptosis induced by Fas (CD95), Bax, caspases, and anticancer drugs. Cancer Res..

[39] Ikeguchi M., Hirooka Y., Kaibara N. (2002). Quantitative analysis of apoptosis-related gene expression in hepatocellular carcinoma. Cancer.

[40] Grossman D., Altieri D.C. (2001). Drug Resistance in Melanoma: Mechanisms, Apoptosis, and New Potential Therapeutic Targets. Cancer Metastasis Rev..

[41] Gradilone A., Gazzaniga P., Ribuffo D., Scarpa S., Cigna E., Vasaturo F., Bottoni U., Innocenzi D., Calvieri S., Scuderi N. (2003). Survivin, bcl-2, bax, and bcl-X gene expression in sentinel lymph nodes from melanoma patients. J. Clin. Oncol..

[42] Fang Y., Eglen R.M. (2017). Three-Dimensional Cell Cultures in Drug Discovery and Development. SLAS Discov..

[43] Antunes N., Kundu B., Kundu S.C., Reis R.L., Correlo V. (2022). In Vitro Cancer Models: A Closer Look at Limitations on Translation. Bioengineering.

[44] Sharma P., Allison J.P. (2015). The future of immune checkpoint therapy. Science.

