# Supplementary material for: Biological Effect of Food for Special Medical Purposes (NutramilTM Complex) on Melanoma Cells in In Vitro Study
Source: Nutrients. 2024 Dec 12;16(24):4287. doi: 10.3390/nu16244287 (PMC11679902; doi:10.3390/nu16244287)
Supplement: Supplementary file 1 [file nutrients-16-04287-s001.zip › nutrients-3339962-supplementary.pdf]

Supplementary material and data

Biological effect of food for special medical purposes (NutramilTM Complex) on melanoma cells in *in vitro* study

Aneta Koronowicz [1*], Katarzyna Krawczyk [1], Aleksandra Such [1], Ewelina Piasna-Słupecka [1], Mariola Drozdowska [1] and Teresa Leszczyńska [1]

[1] Department of Human Nutrition and Dietetics, Faculty of Food Technology, University of Agriculture in Krakow, 30-149 Krakow, Poland

[*]Correspondence: aneta.koronowicz@urk.edu.pl; (A.K.); Tel.: +48-12-6624831

E-mail addresses: aneta.koronowicz@urk.edu.pl (A.K.); aleksandra.such@student.urk.edu.pl (A.S.); katarzyna.krawczyk@student.urk.edu.pl (K.K.); ewelina.piasna@urk.edu.pl (E.P-S.); mariola.drozdowska@urk.edu.pl (M. D.); teresa.leszczyska@urk.edu.pl (T.L.)

Tabel S1. Composition of Nutramil™ Complex as Food for Special Medical Purpose [9]

<table>
<tr><th colspan="5">Nutritional value per 100g of product</th></tr>
<tr><td colspan="4">Energy Value</td><td>1743kJ/ 417kcal</td></tr>
<tr><th colspan="5">Nutrients</th></tr>
<tr><td colspan="4">Total Carbohydrates
- Sugars
- Lactose</td><td>62,5 g
7,2 g
<0,08 g</td></tr>
<tr><td colspan="4">Protein</td><td>15,6 g</td></tr>
<tr><td colspan="4">Total Fat
- Saturated Fatty Acids
- Medium-Chain Triglycerides MCT</td><td>11,7 g
3,5 g
2,4 g</td></tr>
<tr><th colspan="2">Mineral components</th><th colspan="3">Vitamins</th></tr>
<tr><td rowspan="2">Potassium
Calcium
Sodium
Chlorides
Phosphorus
Magnesium
Zinc
Iron
Manganese
Copper
Iodine
Selenium
Molybdenum
Chromium</td><td rowspan="2">485 mg
253 mg
236 mg
222 mg
194 mg
63 mg
2,8 mg
2,1 mg
0,56 mg
278 µg
42 µg
15 µg
14 µg
11 µg</td><td>Fat-Soluble</td><td>Vitamin E
Vitamin A
Vitamin K
Vitamin D</td><td>3,3 mg
222 µg
21 µg
1,4 µg</td></tr>
<tr><td>Water-Soluble</td><td>Vitamin C
Niacin
Pantothenic acid
Vitamin B_2
Vitamin B_6
Vitamin B_1
Folic acid
Biotin
Vitamin B_{12}</td><td>22 mg
4,4 mg
1,7 mg
0,4 mg
0,4 mg
0,3 mg
56 µg
13,9 µg
0,7 µg</td></tr>
</table>
